# The Effects of Ultrasound on the Electro‐Oxidation of Sulfate Solutions at Low pH

**DOI:** 10.1002/cphc.201900346

**Published:** 2019-06-26

**Authors:** Alexander G. Wallace, Patrick J. McHugh, Mark D. Symes

**Affiliations:** ^1^ WestCHEM, School of Chemistry University of Glasgow University Avenue Glasgow G12 8QQ United Kingdom

**Keywords:** electrolysis, electro-oxidation, oxidant, sonoelectrochemistry, sulfate

## Abstract

The electro‐oxidation of sulfate solutions is a well‐established route for the generation of powerful oxidants such as persulfate. Despite this, the effects of simultaneous ultrasound irradiation during this process has attracted little attention. Herein, we investigate the effects of a low‐intensity ultrasonic field on the generation of solution‐phase oxidants during the electro‐oxidation of sulfate solutions. Our results show that at high current densities and high sulfate concentrations, ultrasound has little effect on the Faradaic and absolute yields of solution‐phase oxidants. However, at lower current densities and sulfate concentrations, the amount of these oxidants in solution appears to decrease under ultrasonic irradiation. A mechanism explaining these results is proposed (and validated), whereby anodically‐generated sulfate and hydroxyl radicals are more effectively transported into bulk solution (where they are quenched) during sonication, whereas in the absence of an ultrasonic field these radicals combine with one another to form more persistent species (such as persulfate) that can be detected by iodometry.

## Graphical abstract

**Shaken, not stirred**: The effect of ultrasound on the electro‐oxidation of sulfate solutions is studied as a function of sulfate concentration and current density (see picture), revealing the key role sonochemistry plays in determining product distributions at low sulfate concentrations and low current densities.

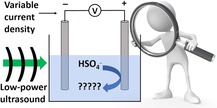

## 1 Introduction

The accumulation of persistent organic pollutants in the natural environment is becoming a matter of increasing concern, on account of the impact these pollutants may have on the environment and human health.^1^ Against this background, so‐called Advanced Oxidation Processes (whereby powerful oxidizing agents are generated in situ and used to oxidize persistent organic pollutants in wastewater streams to less harmful species) have gained traction.^2^ Persulfate (S_2_O_8_^2−^) is one of the oxidizing agents most commonly explored for this purpose,^3^ and indeed its ammonium, potassium and sodium salts are widely used as agents for the oxidative degradation of hazardous organic wastes in the textile, detergent and electronics industries.^4^

Persulfates are prone to decomposition in the presence of moisture and/or at elevated temperatures, and so Advanced Oxidation Process approaches that generate persulfate at the point of use are attractive. Electrochemical oxidation of cold, concentrated aqueous sulfate solutions at high potentials at inert anodes is one such method.^5^ Comninellis and co‐workers first proposed the use of boron‐doped diamond electrodes for this reaction,^6^ and there has since arisen a significant body of literature exploring the mechanism and application of persulfate electro‐generation at boron‐doped diamond anodes.^7^ These studies have established the viability of on‐site electrochemical persulfate production as a strategy for the oxidative degradation of a number of organic pollutants.

However, although persulfate is indeed very a strong oxidant (*E°* for S_2_O_8_^2−^+2 e^−^⇌ 2SO_4_^2−^ lies at +2.01 V vs. SHE),^8^ its rates of degradation of some of the more recalcitrant organic pollutants are limited.^9^ Recently, a strategy circumventing these slow kinetics has emerged, whereby persulfate is activated by application of ultrasound during the pollutant oxidation reaction.^10^ This strategy has been shown to be effective for the degradation (and in some cases, complete mineralization) of various recalcitrant organic pollutants.^11^ The mechanism of this ultrasonic activation is believed to be homolysis of the O−O bond in persulfate,^12^ which generates highly reactive sulfate radicals, (*E°*>+2.5 V vs. SHE).^13^

The application of ultrasound to electrochemical reactions (sonoelectrochemistry) can enhance mass transport in electrochemical reactions,^14^ activate electrode surfaces, accelerate electrode reactions, improve current efficiencies and cause electrochemical reactions to follow different pathways (and sometimes produce different products) to those observed in the absence of ultrasonic irradiation.^15^ However, in spite of the numerous papers dedicated to the electro‐oxidation of sulfate solutions for the synthesis of persulfate and (separately) to persulfate's sonochemical activation, to our surprise, we were unable to find any reports describing the effects of ultrasound on the electro‐oxidation of sulfate solutions. We thus set out to examine how the application of a modest ultrasonic field affected the electro‐oxidation of sulfate solutions at a boron‐doped diamond anode, with a special emphasis on determining how (or if) sonication affected the yield(s) of the oxidant(s) produced and, if so, if any insights into these altered product distributions could be gained.

## 2 Results and Discussion

**Figure 1:**
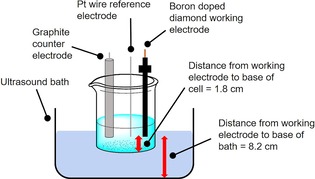
A schematic of the general experimental set‐up used in this study.

Our initial investigations (Table 1, entries 1 and 2) into the effect of ultrasound on the electro‐oxidation of sulfate were conducted in a three‐electrode configuration in 25 mL of 3.6 M (NH_4_)_2_SO_4_ solution in 1 M H_2_SO_4_ (see Figure 1, Experimental Section and Supporting Information for a full description of the experimental set‐up). A 0.071 cm^2^ boron‐doped diamond working electrode, graphite counter electrode and Pt wire reference electrode were used, under an atmosphere of air, in single chamber cells, and with an initial temperature of 18 °C. A current of 0.1 A (current density=1.41 A cm^−2^) was then imposed for 15 minutes. This corresponds to 90 C of charge, or a theoretical yield of 0.466 mmol of persulfate (assuming a 2‐electron process and a 100 % Faradaic efficiency). An ultrasonic field (37 kHz, 443±83 mW, 35 mW cm^−3^) was then applied (Table 1, entry 1) for comparison with the identical experiment in the absence of sonication (Table 1, entry 2).

**Table 1:** The molar and Faradaic yields of oxidants generated under various conditions as described in the main text. In all cases, starting temperatures were 18 °C.

Current density [A cm^−2^]	[SO_4_]^−^ [M]	Sonication at 37 kHz?	Total oxidant yield [μmol]	Faradaic yield for SO_5_^2−^ [%]^[a]^	Faradaic yield for all oxidants [%]^[a]^
1.41^[b]^	4.6	Yes	250±30	3.3±0.3	53±6
1.41^[c]^	4.6	No^[d]^	220±10	1.0±0.1	47±2
0.023^[e]^	1.5	Yes	39±3	5.3±0.2	42±3
0.023^[f]^	1.5	No^[d]^	69±6	2.9±0.2	73±6
N/A^[g]^	4.6	Yes	<2^[h]^	–	–

[a] Faradaic yields calculated on the basis of oxidant formation being a 2‐electron process, as it is for the generation of persulfate, peroxide and Caro's acid. [b] Initial voltage is 3.00 V and final voltage is 3.04 V (vs. Pt). [c] Initial voltage is 3.34 V and final voltage is 3.67 V (vs. Pt). [d] Stirring solutions with a magnetic stir bar at 500 rpm had no noticeable effect on oxidant yields or Faradaic yields. [e] Initial voltage is 2.18 V and final voltage is 2.75 V (vs. Pt). [f] Initial voltage is 2.30 V and final voltage is 2.83 V (vs. Pt). [g] No current applied. [h] Detection limit.

After 15 minutes of electrolysis, the total concentration of oxidant species present in solution in the presence and absence of ultrasound irradiation was gauged using iodometric titration (see Supporting Information for a full description of these methods).^16^ These metrics (Faradaic yield for solution‐phase oxidants=53±6 % with sonication and 47±2 % without sonication) are within the error margin of each other and suggest that sonication at this frequency and power does not impact significantly on the Faradaic yield of solution‐phase oxidants. Noticeable gas evolution was evident at the working electrode during all these electro‐oxidation reactions (both with sonication and without), and it seems likely that the balance of the charge that does not go towards making solution‐phase oxidants is thus consumed in performing the oxygen evolution reaction under these conditions.

The effects of ultrasound‐induced stirring (leading to increased mass transport) were assessed using the calibration protocol developed by Pollet et al.^17^ Hence the limiting current densities obtained from the quasi‐reversible Fe(CN)_6_^3−^/Fe(CN)_6_^4−^ redox couple were obtained in the presence and absence of the applied ultrasound field. These results (see the Supporting Information Figure S1) suggest that limiting current densities are increased approximately 25‐fold under sonication in this set‐up, relative to stirring with a magnetic stir bar at 500 rpm. We also conducted tests to examine the effects of ultrasound‐induced heating. During the 15 minutes of sonication, the temperature of the solution would rise from a starting point of around 18 °C to between 23–24 °C. To isolate the effect of heat from other factors, some control experiments were performed where the reaction vessel was held at a constant 26 °C. In all cases the data obtained were the same (within error) to those obtained without fixing the temperature.

Iodometry is useful for the ready determination of the amount of dissolved oxidant species, but it is somewhat of a blunt instrument in that it cannot distinguish between different oxidants. This is important for our purposes as it is well‐known that aqueous solutions of persulfate can decompose to generate both H_2_O_2_ and SO_5_^2−^ (Caro's acid),^18^ whilst sonication of aqueous solutions can also lead to peroxide formation.^19^ Therefore, we also performed specific colorimetric tests for both H_2_O_2_ and SO_5_^2−^ as described previously by Deadman et al.16b These tests indicated that H_2_O_2_ always accounted for less than 1 % of the total oxidant present in solution, and the Faradaic yields for H_2_O_2_ under sonicated and silent conditions were also within error of each other (0.8±0.2 % with sonication and 0.5±0.2 % without sonication). However, the amount of SO_5_^2−^ that was detected was higher (Faradaic yield=3.3±0.3 % with sonication and 1.0±0.1 % without sonication) and was arguably more significant.

**Figure 2:**
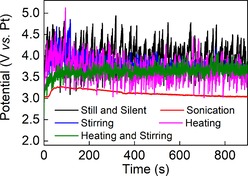
Voltage–time curves for the electrolysis of 3.6 M (NH_4_)_2_SO_4_ in 1 M H_2_SO_4_ at a boron‐doped diamond working electrode and an imposed current density of 1.41 A cm^−2^. Colour‐coding is as follows: unstirred and quiescent starting at 18 °C (black); unstirred starting at 18 °C and sonicated at 37 kHz (red); stirred and quiescent starting at 18 °C (blue); unstirred and quiescent starting at 26 °C (pink); stirred and quiescent starting at 26 °C (green). Stirring was conducted with a magnetic stir bar at 500 rpm.

The voltage‐time profiles for representative electrolysis experiments are shown in Figure 2 (representative cyclic voltammograms under sonicated and silent conditions are shown in Figure S2 in the Supporting Information). Those bulk electrolysis experiments conducted without sonication tended to give rather noisy traces with voltages in excess of 3.5 V (vs. Pt) being required to drive current densities of 1.41 A cm^−2^. However, ultrasonication lowered this voltage demand (typically by several hundred millivolts) and gave voltage‐time profiles that were also less prone to fluctuations (red trace in Figure 2). We ascribe this at least partly to the effectiveness of ultrasound at removing bubbles (generated by the competing oxygen evolution reaction) from the electrode surface,15a which therefore prevents large areas of the electrode from becoming periodically obscured by gas bubbles and gives smoother curves under sonicated conditions.

Previous investigations into electrochemical persulfate production have suggested a link between current density and sulfate concentration in relation to Faradaic yield for persulfate synthesis.^7^ Therefore, we also examined the effect of reducing the current density from 1.41 A cm^−2^ to 23 mA cm^−2^ at the same time as reducing the total sulfate concentration somewhat (from 4.6 M to 1.5 M), in the hope that these less extreme conditions might give greater insight into the reaction products. These results are shown in entries 3 and 4 of Table 1. In agreement with these previous reports, reducing the imposed current leads to a significant increase in the Faradaic yield for such solution‐phase oxidants as can be detected by iodometry (entry 4). This is to be expected on the basis that lower voltages are required to sustain these lower current densities, and hence the competing oxygen evolution reaction is diminished. However, applying a 37 kHz ultrasound field during this electrosynthesis seems to negate the effect of reducing the current density in this manner (entry 3): The Faradaic yield under simultaneous imposition of ultrasonication and a current density of 23 mA cm^−2^ is almost the same as that achieved at 1.41 A cm^−2^. Meanwhile, the control without imposing any current (Table 1, entry 5) shows that essentially no iodometrically‐detectable solution‐phase oxidants are generated by the action of ultrasound alone under these conditions.

The apparently lower yield of solution‐phase oxidants during sonication at an imposed current density of 23 mA cm^−2^ poses the question as to whether sonication disfavours the formation of these oxidants, or whether the oxidants form to the same extent as under quiescent conditions but are then degraded by the ultrasonic field. The latter possibility appears not to be operating here: iodometry on 19 mM aqueous solutions of ammonium persulfate after 0, 15 and 60 minutes of sonication at 37 kHz revealed no diminution of the amount of solution‐phase oxidant. Hence, under these conditions at least, it seems that the ultrasound field does not significantly degrade the solution‐phase oxidants, implying that their lower yield during the sonoelectrochemical protocol in Table 1, entry 3 is probably due to the initial formation of these species being suppressed. We note here that Price and Clifton have previously reported that the application of ultrasound accelerates the rate of persulfate decomposition. It may be that in our case the comparatively low intensity of the ultrasonic field (35 mW cm^−3^, supplied by a bath) compared to this earlier work (where intensities of ∼26 W cm^−2^ were applied using an ultrasonic horn) prevents any noticeable decomposition of the persulfate.^20^ Meanwhile, the very low level of solution‐phase oxidants detected after sonication in the absence of an imposed current (Table 1, entry 5) suggests that the iodometrically‐detectable solution‐phase oxidants are produced essentially entirely by electrochemical processes.

Electrochemical persulfate synthesis from concentrated sulfuric acid at boron doped diamond electrodes at comparatively low current densities has previously been studied by Serrano et al.6b and Davis et al.7c Between them, these authors present a mechanism for the generation of persulfate, peroxide and Caro's acid that proceeds through the generation of both hydroxyl and sulfate radicals (by direct oxidation of water and sulfate respectively at the anode), with the hydroxyl radicals that are formed being themselves able to generate sulfate radicals through the following reactions [Eqs. (1) and (1)]:

(1) $$\mathrm{HSO}{}_{4}{}^{-}+\mathrm{HO}{}^{•}\to \mathrm{SO}{}_{4}{}^{-•}+H{}_{2}O$$

(2) $$H{}_{2}\mathrm{SO}{}_{4}+\mathrm{HO}{}^{•}\to \mathrm{HSO}{}_{4}{}^{•}+H{}_{2}O$$

Whether formed by direct anodic oxidation of sulfate‐containing species or by the mechanisms above, the combination of two sulfate radicals then presents the lowest energy pathway for persulfate generation [Eq. (3)]:

(3) $$\mathrm{HSO}{}_{4}{}^{•}+\mathrm{HSO}{}_{4}{}^{•}\to H{}_{2}S{}_{2}O{}_{8}$$

Meanwhile, peroxide may form by combination of two hydroxyl radicals, whilst peroxomonosulfuric (Caro's) acid may be generated by combination of sulfate and hydroxyl radicals [Eq. (4)]:

(4) $$\mathrm{HSO}{}_{4}{}^{•}+\mathrm{HO}{}^{•}\to H{}_{2}\mathrm{SO}{}_{5}$$

Furthermore, it is known that sulfate radicals can generate hydroxyl radicals by reaction with water at all pHs, although sulfate radicals tend to predominate at acidic pH.^21^

Returning to the data in Table 1, the above equations present an explanation for the difference in solution‐phase oxidant yield observed for entries 3 and 4. In the quiescent experiments (entry 4), production of HSO_4_^.^ occurs in the immediate vicinity of the electrode and hence the local concentration of these radicals is rather high. This then facilitates the rapid dimerization of HSO_4_^.^ to give persulfate according to Equation 3. Applying an ultrasonic field during this process might reasonably be expected to lead to much more effective dispersal of radicals from the vicinity of the electrode into bulk solution and therefore decrease the local concentration of HSO_4_^.^ . This would lead to a lower overall yield of persulfate, but not necessarily a lower yield of Caro's acid, as HSO_4_^.^ dispersed into bulk solution would most likely react with water to produce HO^.^ in the first instance, maintaining a significant flux through Equation 4 in bulk solution. In the case of entries 1 and 2, the higher current densities and sulfate concentrations give rise to a much greater rate of production (and hence local concentration) of HSO_4_^.^ at the anode in both cases, and so the effect of radical dispersal into bulk solution would be expected to be less pronounced than at lower current densities and sulfate concentrations (i. e. as the current density and concentration of sulfate rise, equation 3 comes to dominate regardless of any enhanced mass transport effects).

The above rationale is consistent with the data in Table 1, and in turn predicts that highly reactive radicals generated at the electrode are very rapidly dispersed into bulk solution by the application of the ultrasonic field. Hydroxyl and sulfate radicals have previously been suggested to be the two main species responsible for the degradation of organic pollutants during the sonochemical activation of persulfate^22^ (rather than persulfate itself) on account of their more positive reduction potentials than persulfate. However, the rates of reaction of HSO_4_^.^ and HO^.^ with organic species tend to be approximately an order of magnitude lower than their rates of reaction with themselves or with each other.11i Hence we hypothesised that, if the explanation above was correct, then an organic probe molecule ought to be more rapidly degraded under the conditions in entry 3 of Table 1 than those of entry 4, as under the conditions in entry 3 there would be a greater dispersal of reactive radicals into bulk solution, whilst in the absence of ultrasound the radicals produced would be more likely to react with each other producing (less oxidising) persulfate, hydroxide or Caro's acid.

To test this hypothesis, we investigated the behaviour of the highly‐coloured probe molecule naphthol blue‐black under the conditions shown in entries 3 and 4 of Table 1. Naphthol blue‐black is used widely as a dye in the colouring of fabrics, textiles and soaps, and is extremely stable to both light and heat.^23^ Its discoloration under ultrasound irradiation has previously been studied and it is known that discoloration of the dye corresponds to the start of its oxidative degradation.^24^ Hence there is good precedent that this discoloration is indeed indicative of oxidative degradation of the dye. It is, therefore, an excellent probe molecule for monitoring the effects of (sono)electrochemically‐generated oxidants as it is otherwise very stable and because its partial degradation can be readily measured by monitoring the characteristic absorbance in its electronic spectrum at 620 nm.

**Figure 3:**
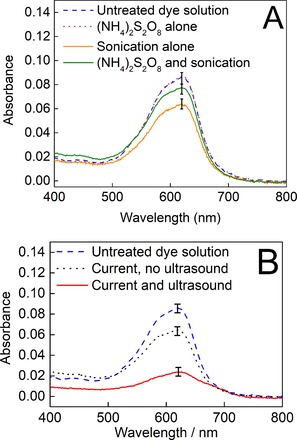
Average absorbances of the dye/electrolyte solutions after 5 minutes of treatment under the following conditions. A) Control stirring at 18 °C (blue dashed line); in the presence of 19 mM (NH_4_)_2_S_2_O_8_ (red dotted line); in the presence of 19 mM (NH_4_)_2_S_2_O_8_ with sonication at 37 kHz (green solid line); sonication at 37 kHz without added (NH_4_)_2_S_2_O_8_ (solid mustard line). B) Control stirring at 18 °C (blue dashed line); under an imposed current density of 23 mA cm^−2^ with stirring (black dotted line); under an imposed current density of 23 mA cm^−2^ with sonication at 37 kHz (red solid line).

Accordingly, solutions of 25 mL of 0.5 M (NH_4_)_2_SO_4_ and 3.2 μM naphthol blue‐black in 1 M H_2_SO_4_ were subjected to a range of conditions and controls, the results of which are summarised in Figure 3 (see also Supporting Information). In Figure 3, each trace is the average of at least three runs and confidence intervals are marked with error bars at the characteristic absorbance of the dye at 620 nm.

Figure 3A shows a series of comparisons between the absorbance of a control solution where the dye/electrolyte solution was left at 18 °C without either electrochemical oxidation or sonication (dashed blue line) and various other (non‐electrochemical) experiments. This control solution returned an absorbance spectrum essentially indistinguishable from that of a freshly‐made dye‐electrolyte solution, implying no background oxidation. The solid orange line shows the absorbance of an otherwise identical dye/electrolyte solution after 5 minutes of sonication at 37 kHz (orange line). Clearly, then, even though iodometry is unable to detect solution‐phase oxidants from sonicated electrolyte solutions, the dye is indeed degraded by ultrasound alone, most likely by sonochemically‐produced hydroxyl and sulfate radicals that are quenched too rapidly to be detected by iodometry. Indeed, the dosimetry protocol developed by Mason et al. (which traps HO^.^ as it forms) suggests that around 6 nmol of hydroxyl radicals are generated under ultrasonic irradiation in our set‐up over a period of 15 minutes (Figures S3 and S4 in the Supporting Information).^25^

Further useful information is provided by deliberately adding the solution‐phase oxidant persulfate to these solutions. Thus, the red dotted line in Figure 3A shows the absorbance of a standard dye/electrolyte solution after stirring at 18 °C for 5 minutes in the presence of 19 mM ammonium persulfate (where a 19 mM solution of ammonium persulfate corresponds to a roughly fifteen‐fold excess in the amount of persulfate that could be produced electrochemically by our methods at this current density in 5 minutes, assuming a 100 % Faradaic yield for persulfate synthesis). This line is indistinguishable from a freshly‐made dye/electrolyte solution and suggests that persulfate on its own does not degrade the dye on this timescale. A similar test using 19 mM H_2_O_2_ also gave an absorbance identical to that of a freshly‐made dye/electrolyte solution. Meanwhile, an experiment in the presence of 19 mM ammonium persulfate as for the red dotted line, but with the application of an ultrasonic field (37 kHz) shows some dye degradation (green line), but intriguingly degradation under these conditions is less apparent than when the dye/electrolyte solution is sonicated without added persulfate. Merouani and co‐workers have previously suggested that excess persulfate may in fact quench the activity of sonochemically‐produced and highly reactive hydroxyl and sulfate radicals through the formation of species such as S_2_O_8_^.−^,11i and this observation agrees with the trend we observe here. Taken together, the results presented in Figure 3A are consistent with a dye degradation mechanism involving hydroxyl and sulfate radicals but in which more stable solution‐phase oxidants such as peroxide and persulfate have no significant effect on dye discoloration.

In Figure 3B, the effect that imposing a current density of 23 mA cm^−2^ for 5 minutes has on the absorbance of the dye/electrolyte solution is shown and compared to the spectrum of a freshly‐prepared dye/electrolyte solution (blue dashed line). When current is imposed in the absence of ultrasound (black dotted line) there is a significant diminution of the dye's absorbance, but there is a far greater decrease in the dye's absorbance when this current density is imposed with simultaneous ultrasonic irradiation at 37 kHz (solid red line), and indeed this combined sonoelectrochemical treatment gave complete discoloration of the dye solution within about 9 minutes.

When the combined effects of ultrasound alone (Figure 3A, orange line) and electrochemical oxidation alone (Figure 3B, black dotted line) are compared to the combined sonoelectrochemical experiment (Figure 3B, red line), there does indeed seem to be synergy between these two processes when they are used together above and beyond what might be expected by simply combining their individual effects on dye discoloration. Given the comparatively low levels of discoloration evident when persulfate is ultrasonically‐activated in situ with the dye (Figure 3A, green line), these data suggest that the synergic effect of the electrochemical oxidation and the ultrasound in this instance is due to the effects of ultrasound on the electrode reactions, rather than activation of electrochemically‐produced persulfate in bulk solution. Hence, these results are also consistent with the hypothesis that the ultrasonic field aids in the rapid dispersal of reactive radicals (generated by electrochemical oxidation) into bulk solution, where they can react with the probe molecule.

**Figure 4:**
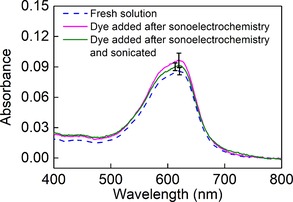
Average absorbances of the dye/electrolyte solutions (at least 3 repetitions for each experiment) after 5 minutes of treatment under the following conditions: 5 minutes of stirring at 18 °C and then dye added and stirred for 5 minutes more (“Fresh solution”, blue dashed line); under simultaneous sonication (37 kHz) and an imposed current density of 23 mA cm^−2^ for 5 minutes, followed by addition of the dye and stirring for 5 minutes more in the absence of current or sonication (pink solid line); under simultaneous sonication (37 kHz) and an imposed current density of 23 mA cm^−2^ for 5 minutes, followed by addition of the dye and sonication (37 kHz) for 5 minutes more in the absence of current (green solid line).

In further support of this supposition, Figure 4 shows the outcome of experiments where a dye‐free electrolyte solution (0.5 M (NH_4_)_2_SO_4_ in 1 M H_2_SO_4_) was subjected to the conditions shown in Table 1, entry 3 for 5 minutes, after which the sonoelectrochemical experiment was terminated. Only then was dye added to the solution to produce a concentration of 3.2 μM naphthol blue black and this solution was stirred for a further 5 minutes (pink line). Likewise, the green line shows what happens in an otherwise identical experiment, but where after the dye has been added the solution is sonicated for 5 minutes. In neither case is any degradation significantly different from that seen in the absence of a preceding sonoelectrochemical process observed. This implies that whatever agent causes the dye discoloration in Figure 3 does not remain in solution long enough to react with a probe molecule subsequently added to the solution. This again points towards highly reactive radicals such as HSO_4_^.^ and HO^.^ as the agents interacting with the probe.

Performing the combined electrochemical‐sonochemical reaction in a two‐chamber electrochemical cell allowed the counter electrode to be placed in its own compartment (separated from the working electrode by a Nafion membrane). This in turn allowed the effects of the cathodic potential at the counter electrode to be separated from the effects of the anodic potential at the working electrode. After 9 minutes of sonoelectrochemical treatment at an imposed current density of 23 mA cm^−2^, the working (anode) electrode compartment had completely discoloured, whilst the counter (cathode) electrode compartment remained blue. This seems to rule out reductive processes as significant contributors to the dye's discoloration.

The experiments above together suggest that the probe is being degraded primarily by sonochemically and anodically‐generated radicals. However, the possibility that the application of ultrasound is simply enhancing mass transport of the dye to the electrode surface (and therefore enhancing the direct electro‐oxidation of the dye) cannot be excluded on the basis of the data presented thus far. Hence a final set of experiments was conducted in which the dye was prevented from accessing the working electrode by means of a cellulose membrane (see Figure S5). This membrane (MEMBRA‐CEL) has a number of rather small pores (nominal molecular weight cut‐off of 3500) that would be expected to impede the movement of the dye (MW=616) but which should constitute considerably less of a barrier to the movement of smaller sulfate and hydroxyl radicals.

When dye was added to the counter electrode compartment of Figure S5 (but not to the working electrode compartment), the diffusion of the dye through the membrane over the course of 30 minutes was very slow, supporting the hypothesis that the membrane slows down the movement of the dye and should prevent it from reaching the working electrode in significant amounts over this time period. Sonoelectrochemical experiments were then conducted as above (current density of 23 mA cm^−2^ in 0.5 M (NH_4_)_2_SO_4_/1 M H_2_SO_4_) and the absorbance of the dye solution monitored after 5 minutes. These results (see Figure S6) suggest that the level of discoloration of the dye is the same (within error) whether a membrane is used to prevent the dye from accessing the anode or not. This in turn suggests that direct electro‐oxidation of the dye at the working electrode is not the main (or at least, not the only) dye discoloration mechanism operating in this system. Instead, these data are consistent with the idea that the dominant probe degradation mechanism is by sonochemically and anodically‐generated radicals, which the ultrasound field then effectively disperses into bulk solution.

## 3 Conclusions

In summary, we have investigated the effects of applying a modest ultrasonic field during the electro‐oxidation of sulfate solutions at a boron‐doped diamond anode on the distribution of products that are obtained. At high current density and high sulfate concentration, the production of solution‐phase oxidants appears to be unaffected by the application of low‐intensity 37 kHz ultrasound in terms of both Faradaic and absolute yields. However, when lower current densities and sulfate concentrations are employed, ultrasound appears to bring about a noticeable diminution in the production of solution‐phase oxidants (as measured by iodometry), relative to non‐sonicated conditions. By employing a suitable probe molecule, we have shown that this apparent decrease in solution‐phase oxidant production in the presence of ultrasound is most likely due to the more rapid transport of the primary products of anodic oxidation generated under these conditions (HSO_4_^.^ and HO^.^ ) from the electrode into bulk solution during sonication. The radicals are therefore rapidly transported from regions of high concentration in the vicinity of the electrode where there is a high chance that they will combine with each other to produce persulfate, Caro's acid and peroxide (all of which can be readily detected by iodometry), with a consequent decrease in the detected levels of most of these species. Moreover, as a direct result of this more rapid transport of highly reactive radicals into bulk solution, the chances that these radicals will react with species in bulk solution (such as the highly‐coloured probe molecule) are increased. Meanwhile, the application of ultrasound itself also leads to the generation of such radicals in bulk solution. The overall effect of the combined sonoelectrochemical treatment is therefore a greater level of probe‐molecule degradation in solution than can be achieved by either the individual sonochemical or electrochemical methods alone. Such insight might prove useful in the development of improved Advanced Oxidation Processes, and further work aiming to investigate the effects of sonoelectrochemistry on the electrolysis of sulfate solutions under a range of other conditions is currently underway in our laboratory.

## Experimental Section

Electrochemical studies were performed in a three‐electrode configuration in single chamber cells unless otherwise stated. The working electrode used was either a boron doped diamond button electrode of surface area 0.071 cm^2^ (supplied by Windsor Scientific Ltd., UK), or a boron doped diamond foil (surface area=0.88 cm^2^) also supplied by Windsor Scientific Instruments. Unless otherwise stated, the reference electrode used was a platinum wire and the counter electrode was a graphite rod (99.9999 %, Sigma Aldrich) with a surface area far greater than that of the working electrode.

For sonication experiments, a Fisher Scientific FB15050 ultrasonic bath (frequency=37 kHz) was employed, always filled with 2.2 L of water. A 100 mL beaker (open to the atmosphere) was used as the reaction vessel, and this was always submerged to the same depth (1 cm) and clamped in exactly the same position in the bath for each experiment. For all configurations, the distance between the working electrode and the base of the cell was 1.8 cm and the distance between the working electrode and the base of the ultrasound bath was 8.2 cm. The volume of solution submerged was 12.5 cm^3^ and the total surface area of the beaker exposed to the bath was 25 cm^2^. Using this set‐up, the consistent temperature rise recorded during the sonication of 25 mL of pure water over 30 minutes (7 °C) could be used to gauge the acoustic power dissipated during sonication as 443±83 mW, by the calorimetric method of Margulis and Mal'tsev.^26^ Using the volume quoted, an ultrasonic intensity of around 35 mW cm^−3^ is obtained.

Further details on procedures and materials can be found in the Supporting Information.

## Conflict of interest

The authors declare no conflict of interest.

## Supporting information

As a service to our authors and readers, this journal provides supporting information supplied by the authors. Such materials are peer reviewed and may be re‐organized for online delivery, but are not copy‐edited or typeset. Technical support issues arising from supporting information (other than missing files) should be addressed to the authors.

Supplementary
