# Supplementary material for: The Effects of Ultrasound on the Electro‐Oxidation of Sulfate Solutions at Low pH
Source: Chemphyschem. 2019 Jun 26;20(22):3134–40. doi: 10.1002/cphc.201900346 (PMC6899799; doi:10.1002/cphc.201900346)
Supplement: Supplementary file 1 — Supplementary [file CPHC-20-3134-s001.pdf]

CHEMPHYSCHEM

Supporting Information

© Copyright Wiley-VCH Verlag GmbH & Co. KGaA, 69451 Weinheim, 2019

The Effects of Ultrasound on the Electro-Oxidation of Sulfate Solutions at Low pH

Alexander G. Wallace, Patrick J. McHugh, and Mark D. Symes*© 2019 The Authors. Published by Wiley-VCH Verlag GmbH & Co. KGaA. This is an open access article under the terms of the Creative Commons Attribution License, which permits use, distribution and reproduction in any medium, provided the original work is properly cited.An invited contribution to a Special Issue on Electrocatalysis.

Index	*Page*
**SI-1**: General experimental remarks	S3
**SI-2**: Electrochemical methods	S3
**SI-3**: Sonochemical methods	S4
**SI-4**: Colorimetric tests	S4
Figure **S1**: calibration with ferricyanide	S6
Figure **S2**: cyclic voltammetry	S7
Figures **S3** and **S4**: dosimetry	S8
Figures **S5** and **S6**: sonoelectrochemistry in the presence of a cellulose membrane	S9

WILEY-VCH

SI-1: General Experimental Remarks: Ammonium sulfate (98+%), sodium metavanadate (96%), potassium iodide (99%) and Naphthol Blue-Black (also known as Amido Black 10B) were obtained from Alfa Aesar. Sulfuric acid (95%) was obtained from Fisher. Ammonium persulfate (≥98%), sodium sulfite (≥98%) and potassium permanganate (≥99%) were obtained from Sigma Aldrich. Hydrogen peroxide (30% by volume) and potassium chloride (analytical grade) were obtained from VWR Chemicals. Titanium oxysulfate (technical grade) was obtained from Riedel-de Haën. Potassium Ferricyanide (ACS grade) was obtained from Acros Organics. All aqueous solutions were prepared using ultrapure grade water (18.2 MΩ cm) obtained from a Sartorius Arium comfort combined water system. UV-Vis spectra were recorded on a JASCO V-670 spectrophotometer using 1 cm pathlength cuvettes.

SI-2: Electrochemical Methods: General: Electrochemical studies were performed in a three-electrode configuration in single chamber cells unless otherwise stated. The working electrode used was either a boron doped diamond (BDD) button electrode of surface area 0.071 cm^2 (produced by chemical vapour diffusion and used as grown after polishing by the supplier, supplied by Windsor Scientific Ltd., UK), or a boron doped diamond foil (surface area = 0.88 cm^2) also supplied by Windsor Scientific Instruments (produced by chemical vapour diffusion and used as grown with the nucleation side exposed). For the ultrasound bath calibration with $[Fe(CN)_6]^{3-}$, a glassy carbon electrode with surface area 0.071 cm^2 (CH Instruments) was used. Unless otherwise stated, the reference electrode used was a platinum wire and the counter electrode was a graphite rod (99.9999%, Sigma Aldrich) with a surface area far greater than that of the working electrode. Working electrodes were polished with alumina powder and washed with water. Both working and counter electrodes were then washed with isopropyl alcohol and deionized water prior to use.

Controlled Current Electrolysis of Sulfate Solutions: Controlled current electrolysis at high current density was carried out using a CHI760d potentiostat version 14.05 or a Palmsens4 potentiostat on a boron doped diamond (BDD) button electrode of surface area 0.071 cm^2. 25 mL of 3.62 M ammonium sulfate solution in 1 M H_2SO_4 was subjected to a current of 100 mA (current density of 1.41 A cm^{-2}) for 15 minutes. This corresponds to 90 C of charge passed. Using Faraday's Law of Electrolysis, a theoretical yield of 0.466 mmol of persulfate (assuming a 2-electron process and a 100% Faradaic efficiency) was thus obtained. This experiment was performed in the presence and absence of applied ultrasonic fields (37 kHz, see below). The effect of the observed temperature rise during sonication was also probed. This data is summarized in Table 1 in the main text (entries 1 and 2).

WILEY-VCH

Controlled current electrolysis at low current density (23 mA cm^{-2}, Table 1, entries 3 and 4) was carried out in a similar manner, except that the working electrode was now a boron doped diamond foil (surface area = 0.88 cm^2) and the electrolyte was 0.5 M ammonium sulfate solution in 1 M H_2SO_4.

All potentials are reported uncorrected for cell resistances, which were found to be around 10 Ω.

Controlled Current Electrolysis in the Presence of Naphthol Blue-Black: 25 mL of 0.5 M ammonium sulfate and 3.2 μM naphthol blue-black in 1 M H_2SO_4 was subjected to a current density of 23 mA cm^{-2} for 5 minutes. Temperatures were initially 18 °C in all cases. When samples were stirred, they were stirred with a magnetic stir bar at 500 rpm. Some of the solutions subjected to controlled current electrolysis were also sonicated at 37 kHz, whilst others were not as described in the main text.

Naphthol Blue-Black Oxidation using Ammonium Persulfate: To 25 mL of 0.5 M ammonium sulfate in 1 M H_2SO_4 was added 0.106 g (0.466 mmol) of ammonium persulfate. The ammonium persulfate fully dissolved. The solution was then made to a concentration of 3.2 μM in naphthol blue-black and left to stir at room temperature for 5 minutes.

SI-3: Sonochemical Methods: A Fisher Scientific FB15050 ultrasonic bath (frequency = 37 kHz) was employed, always filled with 2.2 L of water. A 100 mL beaker was used as the reaction vessel, and this was always submerged to the same depth (1 cm) and clamped in exactly the same position in the bath for each experiment. The volume of solution submerged was 12.5 cm^3 and the total surface area of the beaker exposed to the bath was 25 cm^2. Using this set-up, the consistent temperature rise during sonication of 25 mL pure water over 30 minutes (7 °C) could be used to gauge the acoustic power dissipated during sonication as 443 ± 83 mW.

SI-4: Total Oxidation Determination and Colorimetric Tests

To determine the total amount of oxidants made during electrolysis, iodometric titration was used according to general procedures reported by Deadman *et al.* (B. J. Deadman, K. Hellgardt, K. K. Hii, *React. Chem. Eng.* **2017**, *2*, 462). To the solution to be tested, 200 equivalents of KI were added (relative to the theoretical maximum yield of ammonium persulfate calculated using Faraday's law). This turned the solution dark orange. Sodium thiosulfate of known concentration was then added until the solution became colourless. To aid with detecting the endpoint, starch solution may be added, which reacts with any remaining iodide ions to give a dark blue colour. This method allowed the total amount

WILEY-VCH

of oxidants present in the analyte solution to be obtained on the basis of the amount (and concentration) of the sodium thiosulfate used in the titration.

Determination of Caro's Acid

100 mM vanadyl sulfate solution was prepared by mixing sodium metavanadate with 1:3 diluted sulfuric acid to produce a yellow solution. A molar equivalent amount of sodium sulfite was added forming the blue vanadyl sulfate solution. This solution was then degassed to remove sulfur dioxide and used as below.

1 mol equivalent of $VOSO_4$ (relative to the theoretical maximum amount Caro's acid that could be made based on the charge passed in the electrolysis) was added to the solution to be tested. The vanadyl ion (VO^{2+}) is selectively oxidised by Caro's acid to the pervanadyl ion (VO_2^+). The absorption at 360 nm was measured and was compared to a calibration graph to determine the concentration of pervanadyl. Knowing the concentration of pervanadyl allows the calculation of the concentration of Caro's acid that was in the solution and thus, the amount of Caro's acid made during a particular experiment.

To produce the calibration curve, known amounts of $KMnO_4$ were added to the $VOSO_4$ solution to produce known amounts of pervanadyl, and then the absorption at 360 nm was measured.

Determination of Hydrogen Peroxide

To determine the concentration of hydrogen peroxide present, the analyte solution was mixed with titanium oxysulfate ($TiOSO_4$) (a 1 mole equivalent relative to the theoretical maximum amount of hydrogen peroxide that could be made based on the charge passed). The $TiOSO_4$ solution was made by dissolving $TiOSO_4$ in 2 M H_2SO_4 (aided by sonication). When hydrogen peroxide reacts with $TiOSO_4$, titanic acid is formed, turning the solution yellow. Absorption at 407 nm was taken and compared with a calibration curve to determine the amount of hydrogen peroxide made. To produce the calibration curve, known amounts of H_2O_2 were added to the $TiOSO_4$ solution and then absorption at 407 nm was measured.

WILEY-VCH

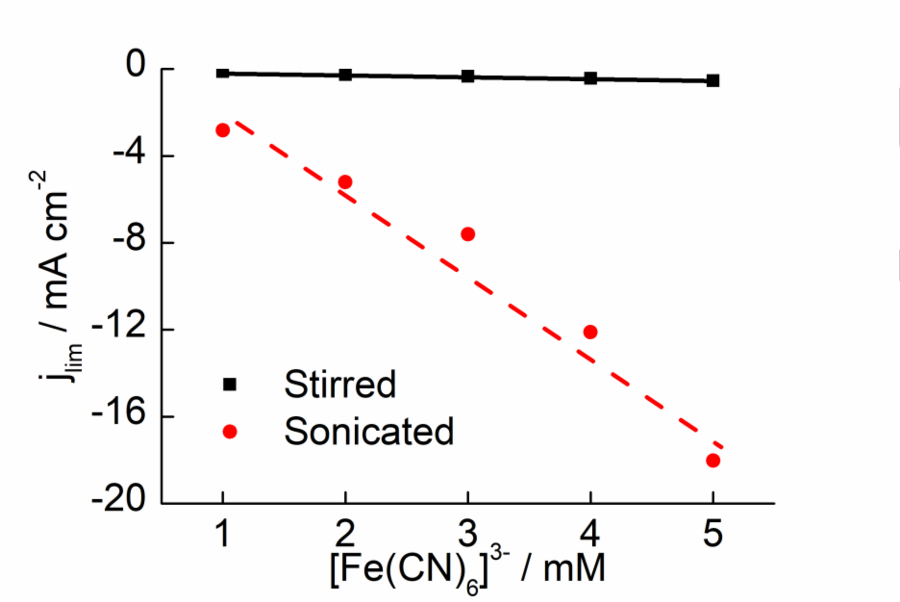

Figure S1: A comparison of the effect of ultrasonically (37 kHz) enhanced mass transport on the limiting current density of the $[Fe(CN)_6]^{3-}$/$[Fe(CN)_6]^{4-}$ redox couple at varying concentrations of ferricyanide (red dashed line). A comparison to stirring at 500 rpm is also shown (black line). The data was obtained by performing linear sweep voltammetry from 0 V to −1 V (*vs.* Pt) at a scan rate of 10 mV/s. The working electrode was a glassy carbon button electrode (area = 0.071 cm^2), the reference electrode was a Pt wire and the counter electrode was a graphite rod. The electrolyte consisted of various concentrations of $K_3[Fe(CN)_6]$ in 1 M KCl.

WILEY

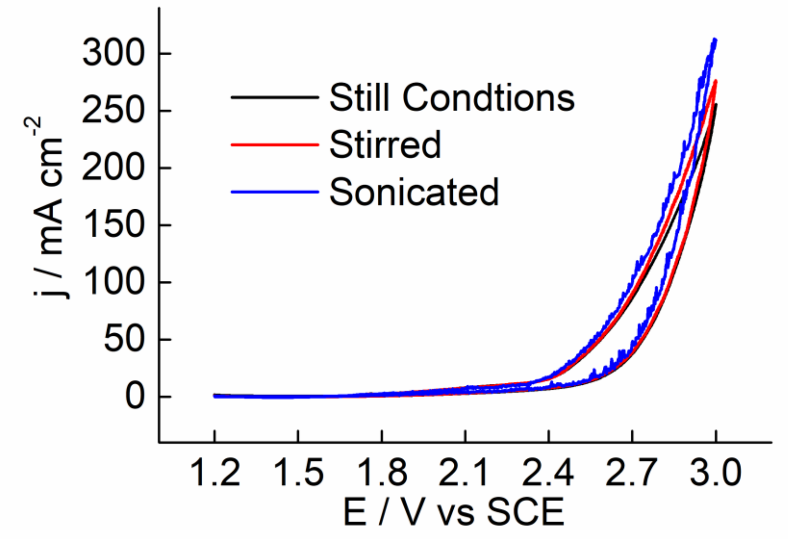

Figure S2: Cyclic voltammetry in 3.6 M $(NH_4)_2SO_4$ / 1 M H_2SO_4 at a scan rate of 50 mV/s. In all cases, a large area graphite rod counter electrode, a boron doped diamond button working electrode (area = 0.071 cm^2) and an SCE reference electrode (saturated KCl, supplied by CH Instruments) were used. Solutions under "stirred" conditions were stirred at 500 rpm with a magnetic stirrer bar.

WILEY-VCH

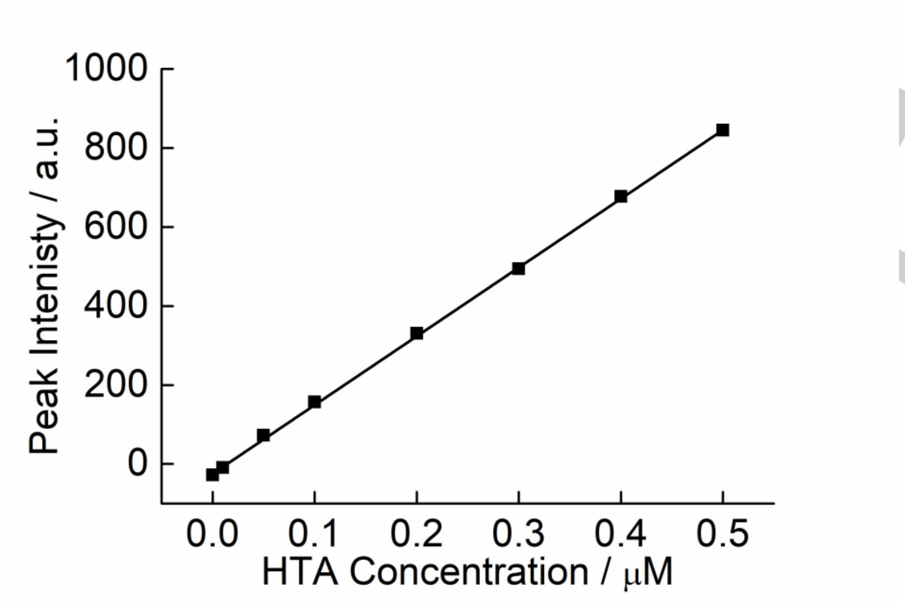

Figure S3: Calibration curve of the fluorescence of 2-hydroxyterephthalic acid (HTA) in aqueous solution (5 mM NaOH / 10 mM phosphate buffer). Fluorimetry conditions: 315 nm excitation wavelength, 330 to 470 nm emission wavelength with a 10 nm excitation slit and a 10 nm emission slit at 600 nm per minute using a Cary Eclipse Fluorescence Spectrophotometer running 1.2(147) software. Peak intensities were measured at 418 nm.

For dosimetry, 25 mL of a 2 mM solution of terephthalic acid in 5 mM NaOH / 10 mM phosphate buffer was then sonicated for 15 minutes in our set-up, and the yield of 2-hydroxyterephthalic acid thus generated by reaction of terephthalate with hydroxyl radicals was measured by fluorimetry as shown below.

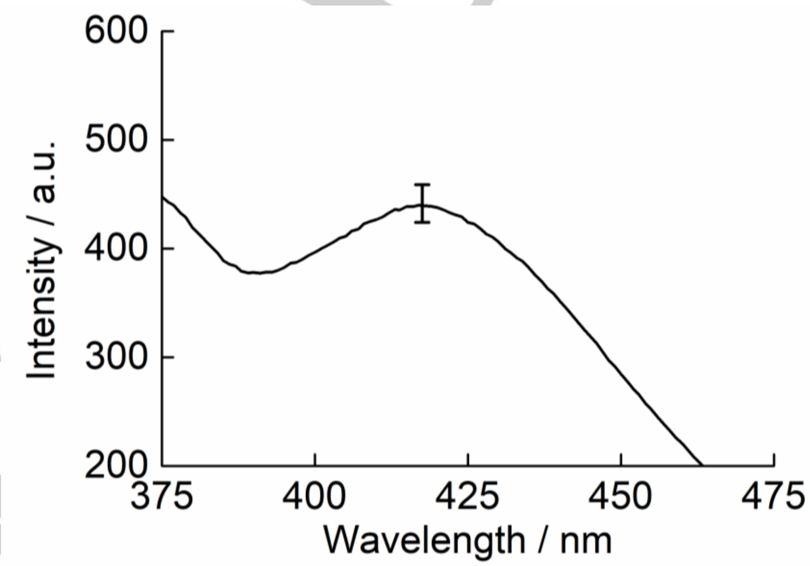

Figure S4: The average (of three repeats) of the fluorescence evident using the dosimetry protocol above. This translates to a concentration of 0.27 ± 0.02 μM 2-hydroxyterephthalic acid, suggesting that at least 6 nmol of hydroxyl radicals are generated during 15 minutes of sonication under our standard conditions.

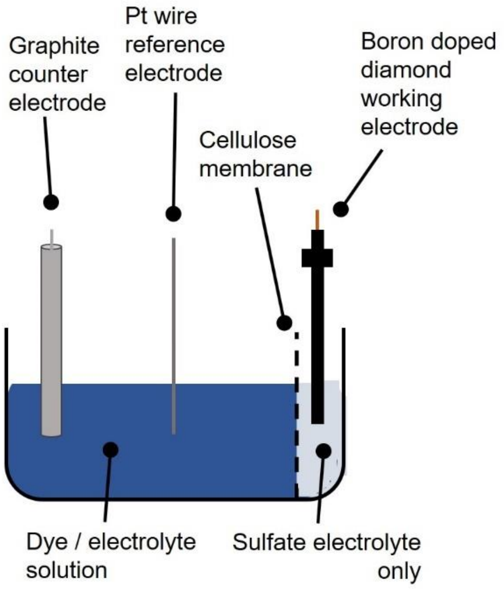

Figure S5: Schematic of the cell set-up with a cellulose membrane preventing the probe molecule from accessing the working electrode directly.

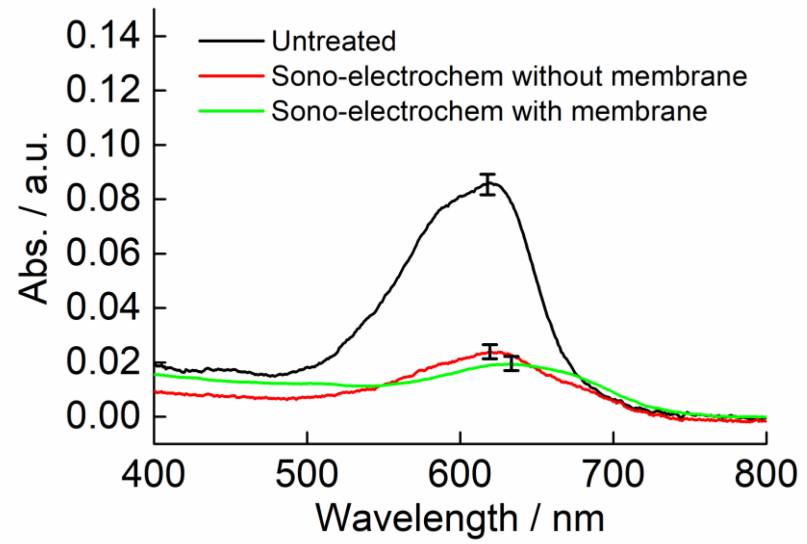

Figure S6: The average (of three repeats) of the absorbance of naphthol blue black when subjected to sonoelectrochemistry as specified in the main text in the absence (red line) and presence of a cellulose membrane (green line). The cellulose membrane prevents the dye from directly accessing the working electrode. Also shown for comparison is a dye-electrolyte solution that was not subjected to any sonoelectrochemical treatment. The sonoelectrochemically-treated samples both with and without a membrane return dye discoloration levels that are the same as each other within error.
